# An Intervention to Support Higher Education Teachers’ Teaching Processes and Well-Being: Protocol for an Intervention Study

**DOI:** 10.2196/65428

**Published:** 2025-06-25

**Authors:** Liisa Postareff, Anna Parpala, Petri Nokelainen, Merly Kosenkranius, Ilmari Puhakka, Laura Pylväs, Heta Rintala, Milla Räisänen, Anna Wallin

**Affiliations:** 1 Unit for Research and Development of Higher Education Pedagogy Häme University of Applied Sciences Hämeenlinna Finland; 2 Faculty of Educational Sciences University of Helsinki Helsinki Finland; 3 Faculty of Education and Culture Tampere University Tampere Finland

**Keywords:** well-being, higher education, teachers, approaches to teaching, guided reflection, mindfulness, arousal, pedagogical awareness

## Abstract

**Background:**

Higher education (HE) teachers are experiencing numerous pressures in their work, such as increased workload, rising student numbers, and declining job resources, making their well-being a crucial issue. Previous studies indicate that adopting a learning-focused approach to teaching (LFT) correlates positively with HE teachers’ self-efficacy beliefs and positive emotions. Moreover, there is growing evidence that mindfulness-based interventions can enhance teachers’ well-being and teaching processes.

**Objective:**

This study aims to describe the design of an intervention developed for higher education teachers to support their teaching processes and well-being. The aim of the intervention is to help teachers reflect on their own teaching, offer tools to use learning-focused teaching methods, and increase teachers’ ability to define the problem and use learning-focused teaching methods using guided reflection and mindfulness-based practices.

**Methods:**

We developed an intervention in which the teachers participate in 4 group meetings and 2 individual guided reflection sessions (before and after all the group meetings). All group meetings and guided reflection sessions were conducted online via Zoom (Zoom Video Communications). Between the group meetings, the participants independently complete self-study assignments and mindfulness-based exercises available on the Moodle platform. In the guided reflection sessions, the teachers reflect on their previously video-recorded teaching situation together with a researcher. During the video-recorded teaching situation, the teacher wears a Moodmetric smart ring measuring the teacher’s arousal level, and episodes with different arousal levels (high, low, and changing) are presented to the participants in the guided reflection sessions. To examine the relations between higher education teachers’ teaching processes and well-being, and the intervention’s effects, we collect longitudinal data before and after the intervention with various methods (eg, experience sampling, interviews, and surveys).

**Results:**

The recruitment of the intervention participants took place in the fall of 2023 from 9 HE institutions in Finland. Altogether, 56 teachers participated in the first part of the intervention (baseline measurement and guided reflection), and 37 participants completed the intervention in spring 2024 by participating in the second guided reflection session and data collection phase. In addition to these 37 participants, 7 teachers who were not able to record their teaching in the second data collection phase but who had still participated in the group meeting and done mindfulness practices, were interviewed about their experiences. The data collection is still ongoing, and additional data will be collected during the academic year 2024-2025.

**Conclusions:**

This study aims to contribute valuable insights into the relations between higher education teachers’ teaching processes and well-being, and how an intervention consisting of guided reflection, group meetings, and mindfulness-based practices may enhance teachers’ awareness of how their teaching is related to well-being and ways to influence it.

**International Registered Report Identifier (IRRID):**

DERR1-10.2196/65428

## Introduction

### Background

Within higher education (HE), the significance of teacher well-being has been highlighted for various reasons, including increased work intensity, consumer-oriented practices, growing student numbers, and declining job resources and stability [1-5]. Supporting teachers’ well-being is important not only for individual teachers’ overall health but also for their students’ learning [6] and well-being [7], as well as for the teachers’ employers from the perspective of teacher attrition and satisfaction [8].

Previous studies on HE teacher well-being have identified several factors that can influence it, such as relationships with colleagues and students, organizational demands and resources, and personal resources. However, considering the characteristics of the teaching profession, it is striking that the role of teachers' pedagogical competence and what kind of teaching processes teachers adopt has been largely neglected in studies exploring HE teachers’ well-being. Previous studies suggest that adopting a student-focused approach to teaching correlates positively with HE teachers’ self-efficacy beliefs [9,10] and positive emotions [11], indicating its potential relevance for their well-being as well.

Thus, this study aims to describe the design of an intervention developed to support HE teachers in their teaching processes and well-being. The intervention is based on guided reflection, group meetings, and mindfulness-based practices with an aim to increase the teachers’ awareness of how their teaching is related to well-being and ways to influence it. To examine the relations between HE teachers’ teaching processes and well-being, and the intervention’s effects, we collect longitudinal data before and after the intervention.

### Approaches to Teaching and Pedagogical Awareness

Approaches to teaching describe what kind of teaching processes teachers adopt, and they mirror the teachers’ pedagogical competence [12-14]. In a learning-focused approach to teaching (LFT), teachers pay attention to the amount and quality of teacher-student and student-student interactions and different methods to activate students’ previous knowledge and their own thinking, in other words, support constructive learning [15]. They flexibly use a wide range of teaching and assessment methods to engage and activate their students according to the situation and reflect on their own actions in the teaching-learning situation. The use of these LFT methods is crucial also in enabling students’ ability to reflect and regulate their learning, relate ideas, and think critically, which in turn have shown to be in relation to students’ well-being and positive experiences in the HE context [16-18].

Moreover, LFT is often associated with teachers’ ability to reflect on their own teaching, leading to increased pedagogical awareness [13]. Previous research among HE teachers has shown that teachers’ awareness of their teaching processes is important for their pedagogical development [13,19]. Reflection is an essential part of teacher development because when thinking deeply about their teaching and experiences, teachers can identify different aspects of the teaching situation and gain greater awareness of their choices [20]. In addition, short pedagogical training has been found to increase HE teachers’ ability to use pedagogical awareness in situational teaching practices [21].

Previous self-report-based research suggests that when teachers learn to adopt more LFT, they feel more positive emotions about their teaching tasks [11] and score lower on scales that measure burnout risk [9]. However, contradictory results have also been found during the COVID-19 pandemic as teachers who typically adopted reflective, learning-focused, and interactive teaching approaches in contact teaching have experienced online teaching as challenging, and thus, been the most stressed [22]. Therefore, our research aims to shed further light on the associations between teachers’ LFT, pedagogical awareness, and well-being.

### Mindfulness to Enhance HE Teachers’ Well-Being

In response to teachers’ increasing stress and high attentional and emotional demands at work, mindfulness-based training has been proposed as an intervention to support and enhance teachers’ well-being and performance as a teacher. Mindfulness is defined as “a kind of nonelaborative, nonjudgmental, present-centered awareness in which each thought, feeling, or sensation that arises in the attentional field is acknowledged and accepted as it is” [23]. Importantly, teachers’ emotions have been shown to influence their students’ emotions and learning [24,25]. Mindfulness may help teachers to be more proactively aware of their daily emotions, and through this, not only protect and enhance their well-being but create a positive classroom climate that supports students’ active learning [26].

The mindfulness-based interventions (MBIs) for teachers primarily focus on enhancing mindfulness, alleviating stress, anxiety, and feelings of burnout, and improving well-being more broadly [27,28]. In support of using MBIs in an educational context, a recent meta-analysis [28] showed that mindfulness-based interventions can increase teachers’ feelings of mindfulness, and alleviate stress, anxiety, and feelings of depression and burnout. Besides investigating how MBIs can alleviate negative mental health outcomes, several recent MBI studies have also focused on the positive aspects of well-being, such as positive affect, life satisfaction, and self-compassion (for systematic reviews, [27,29]). Taken together, a growing body of evidence provides evidence that MBIs have the potential to enhance teachers’ well-being and teaching processes. Therefore, we adopt mindfulness-based practices in our intervention to support teachers’ well-being and ability to use LFT methods in teaching.

### HE Teachers’ Well-Being

In previous studies, HE teachers’ well-being has been conceptualized broadly and diversely, and no widely accepted definition of the concept exists [30-32]. Previous studies have explored it with a predominant focus on stress, burnout, and psychological well-being [33-37]. For instance, [33] recently conducted a review analyzing burnout syndrome among university educators, identifying stress-related causes (eg, work overload) and associated consequences (eg, health issues), alongside factors contributing to variations in stress levels. Similarly, [34] explored antecedents, correlates, and outcomes of faculty burnout, while [35] delved into factors influencing academic psychological well-being, emphasizing adverse effects such as stress, burnout, and negative emotions.

While acknowledging the significance of stress, burnout, and other adverse elements in HE teachers’ well-being, focusing solely on these negative aspects presents a limited view of the phenomenon. Indeed, scholars have advocated for incorporating a positive perspective into the study of well-being to develop a more holistic understanding [36,38]. Thus, in our intervention study, we understand HE teachers’ well-being as encompassing negative components, such as stress, burnout, and negative emotions, as well as positive facets like job satisfaction, work engagement, and positive emotions.

### Aims and Research Questions

The main aim of this protocol study is to describe the design of an intervention developed to support HE teachers’ well-being and teaching processes. We also aim to describe the data collection of longitudinal multi- and mixed methods data that are used to analyze the relationships between HE teachers’ teaching processes and well-being, and for intervention evaluation. To evaluate the effects of the intervention, we aim to answer the following research questions in the forthcoming empirical publications:

- RQ1: what are the associations between the LFT (interaction, constructive approach, and pedagogical awareness) and well-being (ie, burnout, teaching satisfaction, emotions, and stress) on a general level?
- RQ2: what is the impact of the intervention on the relations between LFT, physiological arousal, and emotional states in teaching situations?
- RQ3: what is the impact of the intervention over time (and compared to the control group) on (1) approaches to teaching and pedagogical awareness and (2) well-being (ie, burnout, teaching satisfaction, emotions, stress?

## Methods

### Recruitment and Participants

Participants were recruited from 9 different HE institutions in Finland. The HE institutions were selected based on availability, and the aim was to recruit participants evenly from both the universities and the universities of applied sciences, and from different geographical locations in Finland. The recruitment of HE teachers for the intervention involved advertising through diverse communication channels within participating HE institutions, including intranets, social networking sites, and email lists.

Participants were eligible to participate in the study and the intervention if they were:

- Teaching at a university or university of applied sciences during the academic year 2023-2024.
- Willing and interested in participating in the entire research period carried out during the academic year.
- Willing and interested in participating in group meetings using peer support.
- Able to use the Moodmetric smart ring for 2 weeks during the research period.

The intervention was conducted in Finnish; thus, participating in it required proficiency in the Finnish language. For this reason, participants who were not proficient in Finnish were excluded from participating in the intervention.

### Intervention Design

The aim of the intervention is to help teachers reflect on their own teaching, offer tools to use LFT methods, and increase teachers’ ability to define the problem and use LFT methods using guided reflection and mindfulness-based practices.

The framework for the intervention was initially developed by the first 3 authors. This development process involved designing 3 distinct elements for the intervention: training in LFT methods, mindfulness-based stress reduction (MBSR), and reflection on teaching practices. For the MBSR component, the initial structure and content were reviewed and consulted with a certified MBSR instructor. Following this consultation, the structure and content were revised accordingly. Subsequently, the focus shifted to training in LFT methods. The pedagogical content of the sessions was tested through a pilot involving a group of HE pedagogy experts. Based on the feedback from these experts, the content was further refined and revised. Lastly, the guided reflection sessions were designed to enhance the participants' reflective practices. This development was a collaborative effort involving all the authors, and it was informed by previous research and theories on guided reflection.

The intervention follows the framework of “Constructivist curriculum” [39] in which participants’ reflective learning process is guided by using social interaction, activating previous knowledge, authentic learning experiences, and cooperative dialogue. The learning objectives of the intervention are described as follows: after the training, the participant (1) can teach and guide university students interactively and support their own well-being; (2) recognize the elements of good teaching and guidance and use them in their own teaching, guidance, and teaching development; and (3) become aware of their own pedagogical choices and how to support their own well-being in different teaching situations.

**Figure 1:**
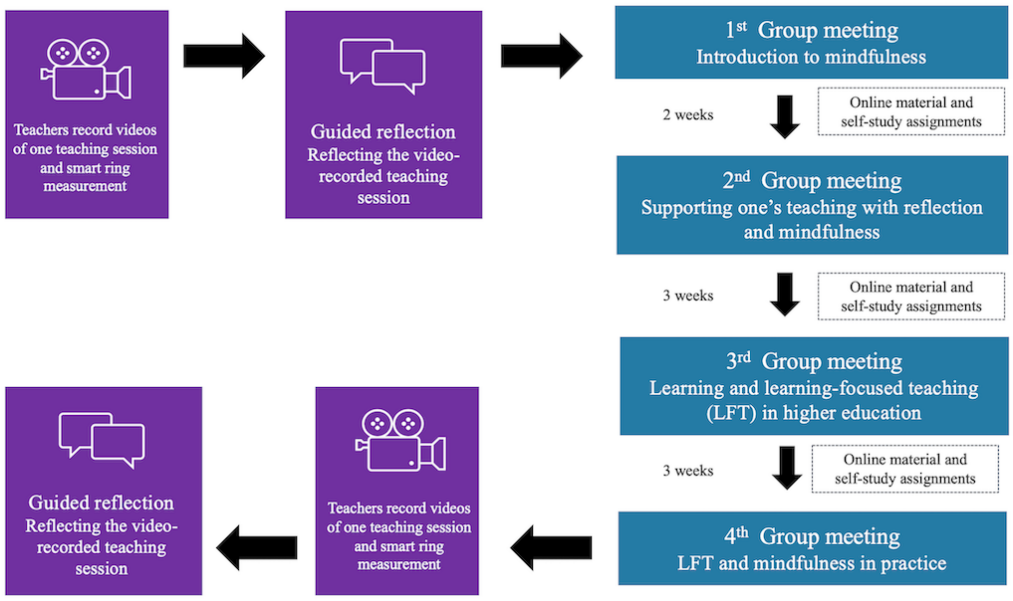
Intervention design.

During the intervention, the teachers participate in 4 group meetings and 2 individual guided reflection sessions (before and after all the group meetings). All the group meetings and guided reflection sessions are conducted online via Zoom (Zoom Video Communications). Between the group meetings, the participants independently complete self-study assignments and mindfulness-based exercises available on the Moodle platform. The participants have 2 weeks to complete the assignments between the first and second group meetings, and 3 weeks between the following group meetings. The first group meeting lasts for one hour and the following 3 group meetings last for 4 hours (Figure 1).

#### Individual Guided Reflection Session

The intervention starts and ends with a guided reflection session conducted via Zoom. Guided reflection has been found to support, for example, student teachers’ ability to implement new methods for improving learning [40] and reflective competencies [20]. It is a process of self-inquiry to enable the practitioner to realize desirable and effective practices with the help of a guide [41,42].

In the guided reflection sessions, the teacher reflects on their previously video-recorded teaching situation together with a researcher. During the video-recorded teaching situation, the teacher wears a smart ring measuring arousal level, and following the critical incident technique [43,44] and stimulated recall interview method, episodes with different arousal levels (high, low, and changing) are presented to the participants in the guided reflection sessions. After watching each episode, the teachers are asked to reflect on what happened during the episodes, what could have influenced their arousal level, and their associated feelings.

#### Group Meetings

All the group meetings are arranged in Zoom. The group meetings follow the Constructivist curriculum [39] and the following elements are included in every session. Every session starts with a discussion of the previous session’s topics, as well as participants’ experiences and questions. Group discussions as well as individual reflection are used during the sessions. At the end of every session, the participants are asked to join a learning discussion on the Moodle learning platform in which they reflect on what was important for them after each session. Mindfulness practices are used during the different sessions.

#### First Group Meeting: Introduction to Mindfulness

During the first group meeting, the teachers are introduced to mindfulness and mindfulness-based practices, and they are given justifications for why mindfulness is selected as a method for the intervention. Mindfulness-based practices have been shown to decrease negative, emotion-focused coping (positive reappraisal and defensive strategies) and increase problem-focused, positive coping strategies (defining the problem, generating alternative solutions, and taking action) [45,46], for example, teachers’ ability to generate solutions and use LFT methods in teaching situations.

In this intervention, we adapt elements of the 8-week MBSR program [47]. The participants are instructed to do mindfulness practices 15 minutes per day during the 8-week intervention. Recent reviews on teachers’ mindfulness interventions [28,29,48] show that while the length of the intervention varies greatly across interventions, mindfulness interventions for teachers are commonly 8 weeks long. Moreover, interventions designed for teachers that are based on MBSR often encourage teachers to engage in shorter self-practices (ie, 10-30 min instead of 45 min per day [48]). In our intervention, teachers are instructed that if they are not able to commit to such intensive practice, they can adjust the effort to their own life situation and opportunities to do the practices. They are instructed to keep a record of how many practices they do. A mindfulness expert is recruited for the intervention to guide the participants throughout the project to do the mindfulness practices. The participants are encouraged to follow mindfulness practices available on the Oiva web page [49] or alternatively, find and complete other mindfulness practices suitable for them. The Oiva web page is a research-based resource that offers over 40 brief mindfulness exercises in the Finnish language that can be easily completed in various settings over the course of a day.

#### Second Group Meeting: Supporting One’s Teaching With Reflection and Mindfulness

The first part of the second session aims to increase teachers’ understanding of the importance of pedagogical awareness. Participants develop their reflection skills and learn how to use reflection to promote pedagogical awareness and how to identify what is stressful and empowering in their teaching. The session includes discussions in small groups, where participants reflect on their experiences of the first guided reflection session. The second part of the session aims to increase and deepen participants’ understanding of what mindfulness is, encourage them to continue practicing mindfulness and reflect on their experiences with mindfulness so far. Participants are introduced to new mindfulness exercises and instructed to first write down pleasant teaching-related experiences for one week’s time and second, unpleasant teaching-related experiences for another week’s time.

#### Third Group Meeting: Learning and Learning-Focused Teaching in HE

The third session focuses on learning and learning-focused teaching in HE. Participants are first introduced to student learning and memorization in HE through interactive learning materials. The participants familiarize themselves with the material first individually and afterward discuss together about the experiences and reflections of the content. Next, the participants will work in small groups to share experiences and thoughts on how to increase students’ understanding and reflection and decrease an unreflective approach to learning a fragmented knowledge base [50] in their own teaching. Secondly, the concept of LFT is introduced to participants through a short presentation. Elements of LFT are also introduced in detail. For example, teacher immediacy is introduced as an important component of creating a safe, learning-focused teaching environment. The session also includes mindfulness practice on self-compassion.

#### Fourth Group Meeting: LFT and Mindfulness in Practice

The focus of the fourth session is teachers’ well-being. Teachers are introduced to teaching-related well-being concepts, such as emotions, self-compassion, psychological flexibility, self-efficacy, stress, and burnout risk. Teachers work in small groups where they share their pleasant or unpleasant teaching experiences and reflect on how they could handle and act in similar situations in the future, in real-life situations. It discusses how learning-focused teaching and mindfulness can support teachers’ well-being in practice, and the teachers brainstorm in small groups on how they can use mindfulness in their teaching.

### Data Collection

**Figure 2:**
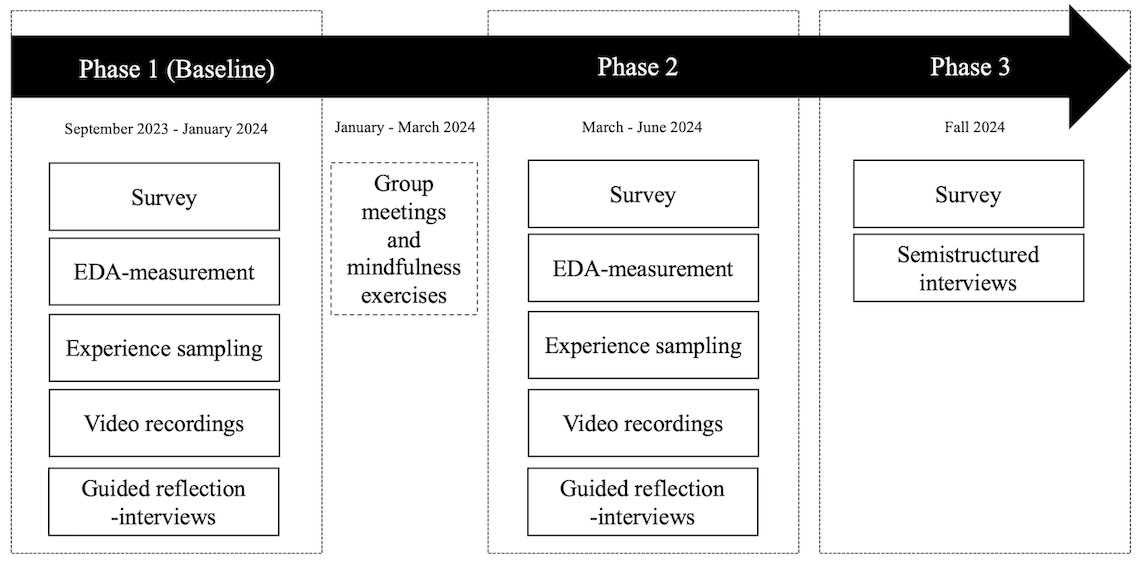
Research design. EDA: electrodermal activity.

To answer the research questions and to evaluate the effect of the intervention, mixed methods data are collected from the participating teachers in 3 phases (Figure 2).

#### Survey

Teachers will respond to the survey 3 times during the study: once before the group meetings (baseline measurement, phase 1) and twice after the group meetings (phase 2 and 3). The survey is completed in Finnish. The survey contains 78 items from 11 scales as described below. Participants answer the survey items on a 5-point scale ranging from 1=completely disagree to 5=completely agree.

#### Approaches to Teaching (HowUTeach)

It [51] is a revised and further-developed version of the Approaches to Teaching Inventory [12]. HowUTeach measures approaches to teaching through 12 items and four dimensions: (1) Interactive approach (3 items, eg, “In my teaching, I create situations where I encourage students to discuss their thoughts and opinions about the topic.”); (2) Transmissive approach (3 items, eg, “The most important goal of my teaching is to deliver what I know to the students.”); (3) Unreflective approach (3 items, eg, “I have trouble understanding how I can help the students to learn”; and (4) Organized approach (3 items, eg, I spend a lot of time preparing my teaching). In addition to the HowUTeach instrument, a new scale labeled as (5) Constructive approach (4 items, eg, “In my teaching, I help students to integrate the knowledge what they have learned in my course to what they’ve learned in other courses”) is used in the research. The scale is developed from the robust and validated instrument HowULearn which measures students’ approaches to learning [17,52]. We modified the scale and items to measure teachers’ strategies to support students’ deep approach to learning. The student version includes items focusing on students’ study strategies such as relating ideas and searching for evidence, and it has been shown to be a reliable scale in previous studies (eg, *𝛼*=.80 in [16]).

#### Teacher Job Satisfaction Scale

It [53] is a 4-item scale that assesses a teacher’s overall job satisfaction (eg, “I enjoy working as a teacher”).

#### Reflection

The 4 items [54] adapted to teaching context (eg, “I sometimes question the way other teachers teach and try to think of a better way”).

#### Mindfulness in Teaching

It [55] was measured with 13 items, 8 items measuring teacher intrapersonal mindfulness (eg, “When I am teaching, I find myself doing things without paying attention”), and 5 interpersonal mindfulness (eg, “I listen carefully to my student’s ideas even when I disagree with them”).

#### Stress

It was measured with a single-item measure of stress symptoms [56] both generally and modified to teaching context (2 items):

Stress means a situation in which a person feels tense, restless, nervous, or anxious, or is unable to sleep at night because his or her mind is troubled all the time. Have you felt this kind of stress these days/related to your teaching?

#### School Burnout Inventory

It [57] was adapted to measure teaching-related burnout through 9 items and 3 dimensions, exhaustion in teaching (eg, “I often sleep badly because of matters related to my teaching”), cynicism toward the meaning of teaching (eg, “I feel that I am losing interest in teaching”), and sense of inadequacy in teaching (eg, “I have often feelings of inadequacy when teaching”). This scale was originally adapted to fit to investigate burnout among HE teachers as part of the HowUTeach project [51], see also the case study by Virtanen and Parpala [10].

#### Self-Efficacy

It was measured with a 4-item scale from the HowUTeach questionnaire [51]. An example item is “I believe I can cope with my teaching tasks.”

#### Self-Compassion

It was measured with a shortened Self-Compassion Scale [58,59]. The shortened 2-factor scale combines positive aspects of self-compassion (ie, self-kindness, common humanity, and mindfulness) into self-compassion factor (eg, “I try to see my failings as part of the human condition.”) and negative aspects (ie, self-judgment, isolation, and overidentification) into self-criticism factor (eg, “I’m disapproving and judgmental about my own flaws and inadequacies.”).

#### Teacher Emotions Scale

This scale [60] measures teachers’ positive and negative emotions through 12 items that cover three dimensions: (1) Enjoyment (eg, “I often have reasons to be happy while I teach.”), (2) Anger (eg, “I often have reasons to be angry while I teach.”), and (3) Anxiety (eg, “I am often worried that my teaching isn’t going so well.”).

#### Anxiety

It is measured with a 6-item Sport Anxiety Scale-2 [61] modified for teaching context. The scale consists of two factors: (1) concentration disruption (eg, “I have trouble concentrating when teaching.”) and (2) worry (eg, I worry that I will make a mistake when I am teaching.”).

#### Ultrashort Utrecht Work Engagement Scale

The scale [62] was adapted to the teaching context to measure teachers’ work engagement. Each dimension of work engagement was measured with one item: (1) Vigor (At my work, I feel bursting with energy”), (2) Dedication (“I am enthusiastic about my teaching job”), and (3) Absorption (“I am immersed in my work as a teacher”).

### Background Information

In the first survey, participants provide information on their demographics (ie, age and gender) and work background (ie, the type of institution they work at, discipline, work experience in HE, pedagogical training, and qualification). In the second survey, participants are asked if they have (1) completed additional pedagogical training and (2) participated in any other mindfulness training during the ongoing academic year.

#### Intervention Evaluation

In the postintervention survey (phase 2), participants additionally answered questions about the intervention process. They are asked to evaluate the effectiveness of the reflection sessions, group meetings, and mindfulness exercises on their (1) general well-being, (2) teaching-related well-being, and (3) development as teacher (understanding teaching and learning processes) on a scale from 1 (“not at all useful”) to 7 (“extremely useful”). They are also asked to evaluate the level of effort they put into participation in the intervention, how demanding they experienced participating in the intervention to be, and their overall satisfaction with the intervention on a 7-point Likert scale. Finally, participants have an opportunity to leave any additional comments about their experience with the intervention.

#### Weekly Mindfulness Reports

During the intervention period, participants receive 9 weekly reminders to report in Webropol (1) how many times and (2) how many minutes they have completed mindfulness exercises during the previous week.

#### Electrodermal Activity Measurement

This study will use a nonintrusive method to collect objective data from teaching situations. To capture participants’ arousal levels during different teaching-related situations, data will be collected via electrodermal activity (EDA) measurements from the participants during 2 one-week measurement periods (phases 1 and 2). The motivation for measuring arousal with EDA (instead of using, eg, heart variability rate) is based on its connection to unconscious emotional responses [63]. We will use an ambulatory biosensor (Moodmetric smart ring; Vigofere Ltd) to measure EDA from the eccrine sweat glands of palmar skin from the hand [64]. It detects skin conductance from the hand wearing the ring. The ring’s algorithm transforms the physiological level of sweat to a within-person standardized value (range 1-100), filtering out bodily movement. Measurement accuracy is close to that of nonambulatory, heavy-duty laboratory equipment [65].

#### Experience Sampling

Participants will report their teaching-related emotional states [66] using the experience sampling method (ESM) [67] during the two EDA measurement periods (phase 1 and 2). The Learning Tracker (LT) smart device application (iOS and Android) [68] is designed to allow participants to self-report their perceived emotions immediately before and after authentic teaching-related situations. Should the participants experience technical issues with the use of the LT application, they can instead respond via a Webropol survey, which contains the same content as the application. During the analysis phase, this longitudinal, discrete data (user-indicated, teaching-related sequences) will be merged with arousal-level data from the smart ring.

#### Video Recordings

All the participants will video-record 2 approximately 60-minute-long teaching situations (during phases 1 and 2). Video data are collected to detect positive and negative teaching episodes and to analyze them further in relation to arousal level, emotions, and LFT. The video recordings of the teaching sessions are done through Zoom, Teams, or with a video camera. The teachers themselves can choose which teaching sessions they want to video-record. The sessions can be face-to-face, synchronous online, or hybrid teaching sessions. If face-to-face or hybrid, the teacher is asked to locate the computer’s camera so that it can observe the teacher. Permission to video-record and use the recordings for research will also be asked from the students using a Webropol survey [69].

Episodes with high arousal levels are identified by synchronizing participants’ EDA level (arousal) data and LT data (emotions) with video data. Episodes are also analyzed through the level of LFT, and how it is manifested in the teaching situations (the use of student-activating methods). Following the coding approach of [70], high arousal episodes are segmented based on LFT and emotional states, and they are analyzed using patterns and temporal order.

#### Guided Reflection—Interviews and Semistructured Interviews

Teachers’ guided reflection sessions will be used as qualitative data. The guided reflection-interviews are semistructured in nature and guided by a list of open-ended questions. In addition to focusing on reflecting on the video-recorded (high, low, and changing) arousal episodes, the teachers are asked general questions related to describing the video-recorded teaching session and their overall teaching experiences. The interview questions are the same in phases 1 and 2, with the exception that in phase 1, the teachers are asked to recall empowering and challenging teaching situations, whereas in phase 2, they are asked to describe themselves as teachers and what they regard as important. In phase 2, the teachers are also asked questions related to intervention evaluation.

Semistructured interviews will also be conducted with voluntary participants in fall 2024 (phase 3) to qualitatively assess the long-term effect of the intervention on their teaching processes and well-being. Before the interviews, participants will receive an individualized summary of their survey results via email. This summary will include their personal results from surveys conducted in phases 1 and 2, focusing on scales measuring approaches to teaching, stress, and teacher job satisfaction. During the interview, teachers will be asked to reflect on their survey answers, including any thoughts the results provoke and, if changes are observed in their answers between phases 1 and 2, what they believe might have contributed to them. In addition, teachers are asked to evaluate the significance of the intervention and discuss more generally the factors they perceive as influencing their well-being as teachers.

### Analytical Approaches

Given the risks of both recruiting a sufficient number of participants and the potential for attrition (typical of longitudinal studies), we designed the study so that statistical analyses could be conducted using a within-subject design (without a control group). The required sample size for the within-subject design analyses is 33 participants, based on conventional power levels set to .80 (*β*=.20) and an α level of .05. However, we also aim to collect a representative control group data and perform analyses using a between-subjects design. In qualitative research, sample size can be flexibly determined based on the richness of the data and saturation [71], one guideline being that 12 interviews are sufficient for thematic saturation [72].

The survey data will be merged with the longitudinal discrete ESM data (user-indicated, teaching-related sequences) from the LT application and continuous EDA data from the smart ring. The resulting panel data will be analyzed using nonlinear and linear mixed-effects methods in the R statistical computing environment [73] using the nlme [74] and brms [75] packages. In the analyses, time-invariant survey data and time-variant ESM and EDA data serve as Level 1 indicators, while the clusters (teachers) are at level 2.

All interviews are recorded, pseudonymized, and transcribed for analysis. ATLAS.ti (Lumivero, LLC) software is used to aid the data analysis. The analysis will be conducted in researcher teams, and the data will be analyzed by more than one person. The research team members conducting the analysis will frequently meet to discuss the analysis and solve any discrepancies. The intercoder agreement is calculated when considered suitable. The qualitative interview data will be analyzed with both inductive and deductive qualitative content analysis, taking into account both latent and manifest content [76,77]. The applied approaches of the qualitative analysis will take different forms in the published articles.

### Ethical Considerations

The research follows strictly the ethical guidelines formatted by the Finnish National Board on Research Integrity [78]. The research was also ethically reviewed in November 2022 by the Human Sciences Ethics Committee of the Helsinki Region Universities of Applied Sciences with a positive decision (statement number: 20/2022) [79].

Prospective participants were guided to the project website for comprehensive details about the intervention and access to a Webropol survey to sign up for the study. Upon providing their contact information, researchers contacted them to agree on the next steps of participation. Before the intervention, each participant met with a member of the research team either online via Zoom or in-person to sign an informed consent form and receive instructions on how to record one of their teaching sessions, sign up for the experience sampling application, and use the smart ring needed for the measurement period. Participation was voluntary and participants could withdraw at any time. A privacy notice is prepared for research participants to inform them about how their personal and other data will be collected, used, stored, and protected. All participant data are handled and combined using assigned ID codes. Data are maintained accordingly for analysis and verification purposes for 5 years after the project’s end. When storing the data, all identifiers will be removed, and the data anonymized (data files containing personal data and participant IDs are erased). Participants did not receive compensation for their involvement; however, they were awarded a certificate of attendance upon completing the intervention upon request.

## Results

The data collection is ongoing. The recruitment of the intervention participants took place in the fall of 2023. As of June 2024, the intervention participants completed phase 2 of the data collection. Phase 3 data were collected in autumn 2024. Moreover, to evaluate the effectiveness of the intervention on HE teachers’ LFT and well-being - compared to teachers who do not undergo the intervention - we aim to collect survey data from teachers working in the same HE institutions as the intervention participants. These teachers will form a control group. The additional survey data will be collected during the academic year 2024–2025.

Altogether, 68 teachers committed to participating in the baseline data collection in fall 2023. Of these 68 teachers, 64 (94%) answered the first survey, and 61 (90%) provided a video recording of their teaching. As part of the intervention, 56 (82%) teachers participated in the first individual guided reflection session. In spring 2024, after participating in the group meetings and completing mindfulness-based practices, 37 (54%) participants completed the intervention by participating in the second guided reflection session. This also marked the completion of the second data collection phase. In addition, 7 (10%) teachers who were not able to record their teaching but had still participated in the group meetings and done mindfulness practices answered the second survey and were interviewed about their experiences. In phase 3, 43 (63%) participants answered the third survey, and 19 (28%) of them also participated in the semistructured interviews.

## Discussion

### Overview

Our intervention study is expected to shed light on how a relatively brief intervention for HE teachers may yield positive effects on pedagogical processes and well-being. Recent research shows that even a short pedagogical training may contribute to HE teachers’ pedagogical development and adaptation of LFT methods [21]. Especially the social aspect of reflection (ie, peer support and feedback) has been identified as an important component of the professional development of HE teachers [80]. In our intervention, in line with the framework of the constructivist curriculum [39], we use guided reflections and group meetings to stimulate the social aspects of HE teachers’ reflection. Moreover, we combine this with 8-week long practice of mindfulness, which is expected to provide teachers a proactive way to become more aware of their present emotions and thoughts, and through this contribute to students’ learning and their own well-being [26]. By examining positive well-being outcomes (eg, work engagement and job satisfaction) in addition to deficit-based mental health outcomes (eg, stress and burnout), we also address the call for future MBIs to focus more on positive well-being constructs, such as work engagement [27].

In this intervention study, we combine research on teaching processes and well-being—2 research areas that have previously been explored separately. By doing so, we contribute to a deeper understanding of how pedagogical competence, along with the ability to cope with emotions and stress during teaching, will impact HE teachers’ well-being. Moreover, as previous research has largely focused only on schoolteachers’ well-being, this study will provide important new knowledge about HE teachers’ well-being. Importantly, the intervention study allows for a comparison of the short- and long-term impact of an intervention focusing on increasing LFT on teachers’ well-being (both physiological and self-reported). The longitudinal multi- and mixed methods design of the study provides valuable information concerning the effects of a professional development program promoting reflection and mindfulness on teaching processes and well-being.

There are several limitations that are worth considering when interpreting the design of this intervention study. First, the intervention includes many elements that place a burden on teachers, such as recording their own teaching, learning to use LT and smart ring, responding to a smartphone application, participating in guided reflections, and attending multiple group meetings. Thus, as the participants were self-selected, it can be assumed that the teachers who chose to participate in the intervention were exceptionally motivated and possessed certain personal characteristics, such as an interest in mindfulness and self-development, compared to other teachers. However, considering the benefits gained from the intervention, self-selectors have been found to benefit more from interventions compared to non–self-selected participants [81].

Second, one methodological limitation is the reliance on self-report data. This is particularly problematic from the perspective of social desirability bias, meaning that participants may, for example, overestimate the effects of the intervention or respond to questions in a socially desirable manner. In the video-recorded stimulated recall -interviews the teachers relive the video-recorded teaching session, and this viewing-relieving process can be very stressful, and teachers’ insecurity, self-confidence, and personal perceptions can influence what they are willing to mention during the interviews. Thus, to control the teachers’ anxiety, a judge-free interview atmosphere is important [82].

Third, this intervention study includes only HE teachers. However, it would have also been useful to include the students' perspectives to retain a more holistic view. Thus, adding student perspectives could be considered when designing similar interventions in the future. Moreover, since discussing one’s feelings and well-being is easier in one’s native language, only teachers proficient in Finnish were eligible to participate in the intervention. However, there is a need to implement similar interventions in the future for migrant and international teachers who are not fluent in Finnish, considering that they encounter numerous challenges that potentially challenge their well-being, professional identity, and self-efficacy in teaching.

### Conclusions

Overall, our study aims to contribute valuable insights into the relations between HE teachers’ teaching processes and well-being, and how an intervention consisting of guided reflection, group meetings, and mindfulness-based practices may enhance teachers’ awareness of how their teaching is related to well-being and ways to influence it. The implications of this study may contribute to the development of future relatively brief interventions and courses to support HE teachers’ pedagogical awareness and well-being.

## Abbreviations

EDA: electrodermal activity
ESM: experience sampling method
HE: higher education
LFT: learning-focused approach to teaching
LT: Learning Tracker
MBI: mindfulness-based intervention
MBSR: mindfulness-based stress reduction

## Data Availability

Data sharing is not applicable to this article as no data sets were generated or analyzed during this study.
